# A Special Virtual Issue Celebrating the 2022 Nobel Prize in Chemistry for the Development of Click Chemistry and Bioorthogonal Chemistry

**DOI:** 10.1021/acscentsci.2c01430

**Published:** 2022-12-05

**Authors:** Carolyn Bertozzi

In the late 1990s, my fledgling lab at UC Berkeley contemplated a new type of chemistry that could be performed in living systems—cells, model organisms, human patients—for various applications in molecular imaging, particularly of cell surface glycans, as well as drug development and delivery. It would still be a few years before we coined the term “bioorthogonal chemistry” to describe the constellation of attributes such chemical reactions would need to possess. The components should be mutually and selectivity reactive, and neither interact nor interfere with the biological system at hand. We drew analogies to the remarkable selectivity of monoclonal antibodies, which can pick out their target from a complex milieu and form a high-affinity noncovalent complex *in vitro* or *in vivo*. In a perspective from those early days, we pondered:

If the same specificity could be distilled into a single covalent linkage, a small reactive molecule could be used as a targeting device and thus create new opportunities for the development of therapeutic and diagnostic strategies...Such a reaction might enable the assembly of complex structures from simple components and the chemical targeting of biomolecules or even whole cells in vivo, opening the door to numerous opportunities in biotechnology and biomedical research.^1^

A few years later, we realized this radical notion with the development of the Staudinger ligation of azides with triarylphosphines,^2^ the inaugural bioorthogonal reaction, and demonstrated its use on cultured cells and in live animals.^3^ The gates were flung open, and a new field was born.

Meanwhile, Prof. Barry Sharpless at The Scripps Research Institute was contemplating the need for highly selective and reliable reactions that could conjoin two partners regardless of surrounding functionality. Brilliantly branded with the name “click chemistry”, such reactions would enable the assembly of complex molecules from simple building blocks with high efficiency and without the contortions imposed by protecting groups.^4^ In 2002, what we now know as the pinnacle of click chemistry—the Cu(I)-catalyzed cycloaddition of azides and terminal alkynes—was reported independently by Prof. Morten Meldal from the University of Copenhagen^5^ and Prof. Sharpless in collaboration with Prof. Valrey Fokin, then also at Scripps.^6^ The addition of the Cu(I) catalyst transformed the reaction from one with no hope of product formation on the time scale of years to one that produced a clean, high-yielding triazole adduct within minutes. The power of this reaction as a bioconjugation tool was made immediately evident by Prof. Meldal’s particular application: linking together peptides and sugars on the solid phase. Seemingly overnight, click chemistry transformed the fields of chemical biology and medicinal chemistry, enabling the assembly of richly functionalized molecules that were previously impossible to construct. Prof. Sharpless often refers to this transformation as coming from an “alien place”, an unexpected and still somewhat mysterious process with unmatched power even today, 20 years after its serendipitous discovery.

At this point, the trajectories of bioorthogonal and click chemistries converged, as each concept embodied elements that were relevant to the other—supreme selectivity in the face of diverse surrounding functionalities, for example. But bioorthogonality imposes additional constraints, in that the reagents also must be nontoxic in living systems, and the reactions need to go fast in water. On this last point, the Staudinger ligation fell short, being rather sluggish at physiological temperatures. Thus, we were actively pursuing faster reactions with azides and took note when the Cu(I)-catalyzed reaction with terminal alkynes hit the press. However, we knew the Cu(I) catalyst would be toxic to cells and animals, thus limiting its use to synthetic or *in vitro* biological applications. To capitalize on azide–alkyne cycloaddition chemistry for use in living systems would require a different mechanism of rate acceleration.

We ultimately achieved this using ring strain, building from early work of Wittig and Krebs who reported that cyclooctyne, the smallest stable cycloalkyne, reacted vigorously with azides at room temperature.^7^ Functionalized cyclooctynes turned out to be perfect reagents for what we called the strain-promoted azide–alkyne cycloaddition (abbreviated SPAAC)^8^ and colloquially referred to as “Cu-free click chemistry”.^9^ This bioorthogonal click reaction opened the door to *in vivo* imaging applications^10^ and has become the new standard, displacing the Staudinger ligation, for use in living systems.

Both Cu(I)-catalyzed and Cu-free click chemistries are now mainstays of chemical biology with applications far beyond our own laboratories’ activities. They are used for imaging, profiling, and discovery of literally every biomolecule class–proteins and their posttranslational modifications, nucleic acids, glycans, lipids, metabolites, and targets of small molecule drugs. They are also widely deployed to construct small molecule and biologic pharmaceuticals with newfound precision. These include antibody–drug conjugates and vaccine formulations that are either FDA-approved drugs or undergoing human clinical evaluation. New drug delivery strategies have been enabled with bioorthogonal chemistry, as showcased by an ongoing clinical trial wherein the reaction—in this case the tetrazine ligation developed by Profs. Joseph Fox^11^ and Neal Devaraj^12^—proceeds within the bodies of cancer patients.^13^ And the utility of click chemistry seems endless, extending beyond chemical biology and medicinal chemistry to many other fields such as materials science.

In recognition of the impact of click and bioorthogonal chemistries, the Chemistry Nobel Committee of the Royal Swedish Academy of Sciences awarded the Nobel Prize in Chemistry to Prof. Sharpless (his second!), Prof. Meldal, and me this past October. As Editor-in-Chief of this journal, it is my distinct honor to celebrate the moment with a collection of science enabled by our collective work. To assemble this virtual issue, we asked the Editors of journals across the ACS publications portfolio for contributions, and the response was overwhelming. We culled the list to this representative collection that reflects a wide range of applications, mechanistic studies, and further reaction developments. These authors leveraged the unique qualities of click and bioorthogonal chemistries in creative and exciting new ways. The field has indeed taken on a life of its own, and our editorial team at *ACS Central Science* is proud to host this issue.
